# In vitro neuronal network activity in NMDA receptor encephalitis

**DOI:** 10.1186/1471-2202-14-17

**Published:** 2013-02-05

**Authors:** Sabine U Jantzen, Stefano Ferrea, Claudia Wach, Kim Quasthoff, Sebastian Illes, Dag Scherfeld, Hans-Peter Hartung, Rüdiger J Seitz, Marcel Dihné

**Affiliations:** 1 Department of Neurology, LVR-Klinikum Duesseldorf, Bergische Landstrasse 2, 40629, Duesseldorf, Germany; 2 Department of Neurology, Medical Faculty, Heinrich-Heine University Duesseldorf, Moorenstrasse 5, 40225, Duesseldorf, Germany; 3 Institute of Clinical Neuroscience and Medical Psychology, Medical Faculty, Heinrich-Heine, University Duesseldorf, Universitaetsstr. 1, 40225, Duesseldorf, Germany

**Keywords:** Autoimmune disease, Encephalitis, Neuropsychological assessment, Paraneoplastic syndrome, Anti-NMDA-antibody

## Abstract

**Background:**

Anti-NMDA-encephalitis is caused by antibodies against the N-methyl-D-aspartate receptor (NMDAR) and characterized by a severe encephalopathy with psychosis, epileptic seizures and autonomic disturbances. It predominantly occurs in young women and is associated in 59% with an ovarian teratoma.

**Results:**

We describe effects of cerebrospinal fluid (CSF) from an anti-N-methyl-D-aspartate receptor (NMDAR) encephalitis patient on in vitro neuronal network activity (ivNNA). In vitro NNA of dissociated primary rat cortical populations was recorded by the microelectrode array (MEA) system.

The 23-year old patient was severely affected but showed an excellent recovery following multimodal immunomodulatory therapy and removal of an ovarian teratoma. Patient CSF (pCSF) taken during the initial weeks after disease onset suppressed global spike- and burst rates of ivNNA in contrast to pCSF sampled after clinical recovery and decrease of NMDAR antibody titers. The synchrony of pCSF-affected ivNNA remained unaltered during the course of the disease.

**Conclusion:**

Patient CSF directly suppresses global activity of neuronal networks recorded by the MEA system. In contrast, pCSF did not regulate the synchrony of ivNNA suggesting that NMDAR antibodies selectively regulate distinct parameters of ivNNA while sparing their functional connectivity. Thus, assessing ivNNA could represent a new technique to evaluate functional consequences of autoimmune encephalitis-related CSF changes.

## Background

Anti-N-methyl-D-aspartate receptor (NMDAR) encephalitis is a recently described disorder characterized by psychosis, epileptic seizures, inflammatory cells in cerebrospinal fluid (CSF) and NMDAR-binding antibodies detectable in serum and CSF [1]. Initially described as a paraneoplastic disease that mostly affects young women with ovarian teratomas [1-3], latest studies report the occurrence of anti-NMDAR encephalitis in older patients and children with or without the presence of a tumor [2,4,5]. Impaired consciousness, obtundation and autonomic disturbances can be lifethreatening. Treatment recommendations include removal of the tumor and immunosuppressive strategies like administration of methylprednisolone, cyclophosphamide, immunoglobulins, plasma exchange or rituximab. While 75% of treated patients have been found to recover completely or retain only mild deficits, 25% remain severely disabled and few even die [3]. Interestingly, clinical improvement seems to correlate with a reduction of NMDAR antibody titers in CSF and serum [2].

The diagnostically defining anti-NMDAR antibodies bind to the surface of CNS neurons. It was shown that the binding epitope is part of the NR1-subunit of the NMDAR on postsynaptic dendrites in the forebrain and hippocampus [1-3]. After capping of NMDARs by anti-NMDAR antibodies, NMDARs are internalized, and consecutively, synaptic NMDAR cluster density decreases. This process is reversible and cell death is not observed [6]. Electrophysiological investigations showed that patients’ CSF containing anti-NR1 antibodies decreased NMDAR-mediated spontaneous miniature excitatory post synaptic currents (mEPSCs) while AMPA-mediated mEPSCs remained unaltered [6]. Thus, anti-NR1 antibody-mediated internalization of NMDARs directly and specifically affects NMDAR-mediated currents. GABA receptors were not impacted demonstrating a specific hypo-functional effect of patients’ CSF on NMDARs. This concept is in line with the observation that NMDAR dysfunction contributes to several neuropsychological disorders like psychosis and that numbers of NMDARs are regulated in response to neuronal activity [7]. Notably, ketamine acts as an NMDAR antagonist and induces behavioral abnormalities similar to the symptoms found in NMDAR encephalitis [8,9]. To investigate effects of NMDAR antibodies containing CSF on a more complex system whose functionality depends on different neurotransmitter-specific neurons like glutamatergic and GABAergic neurons as well as excitatory and inhibitory synapses which are all supported by astrocytes, we made use of a mixed, dissociated neural population generated from rat cortex whose electrophysiological activity was detected by microelectrode arrays (MEAs) [10]. After approximately 3 weeks in culture, neural populations had developed a dense neurite network, the prerequisite for the observed spontaneous population bursting. MEAs are composed of multiple, spatially distributed extracellular electrodes that are able to measure spike and burst activity at different sites of the population.

Here, we report that CSF from a young woman with anti-NMDAR encephalitis suppressed in vitro-neuronal network activity in the acute stage of the disease and that this effect was reversible when the patient recovered upon removal of an ovarian teratoma and subsequent immunosuppression. Network suppression became manifest in significantly reduced spike and burst rates while network synchrony was preserved. Interestingly, this reaction pattern is also observed when functional neuronal networks are exposed to a specific NMDA receptor antagonist [10].

### Case

**Figure 1:**
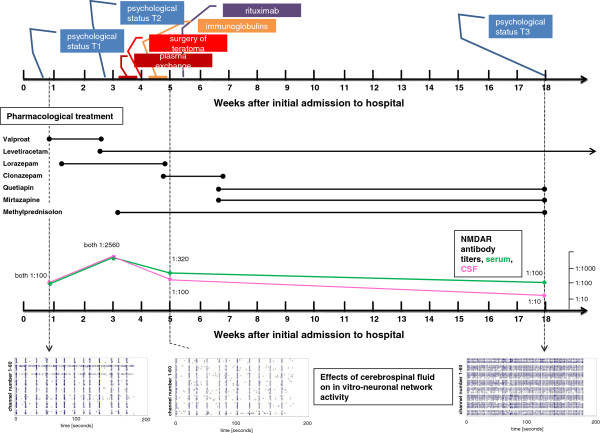
**Time course of disease.** Therapeutic procedures, CSF antibody titers and representative spike raster plots (x-axis: recording time in seconds; y-axis: channel number) of MEA experiments are given in relation to the time after initial hospitalization of the patient. Time points (T1-T3) of psychological tests are given.

**Figure 2:**
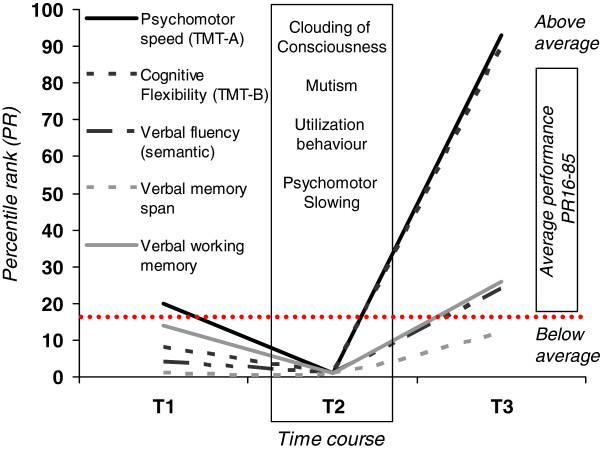
**The neuropsychological course.** At time point 1 (T1) the patient suffered from acoustic illusions, incoherence of thought, psychomotor agitation, mood instability and speech dysfunction (perseveration and neologisms). Mini-Mental-Status-Examination (MMSE: 22/30) showed clinically relevant cognitive impairment. Psychomotor speed assessed by the Trail-Making-Test-A (TMT-A) was average, cognitive flexibility/task switching (TMT-B), verbal memory span, working memory and semantic category verbal fluency were below average (percentile rank < 16). At T2, formal neuropsychological testing was not feasible due to clouding of consciousness, mutism and pronounced psychomotor slowing. Moreover, the patient displayed undirected utilization behaviour suggestive of severe frontal lobe dysfunction. At T3, neuropsychological functioning was considerably improved. MMSE was inconspicuous (30/30). With the exception of discrete impairment regarding verbal memory span, formal neuropsychological testing revealed above average psychomotor speed (TMT-A) and cognitive flexibility (TMT-B), average working memory and executive functioning as measured by semantic category verbal fluency. Interestingly, phonemic verbal fluency, which is considered to involve frontal cognitive control even more than semantic category tasks, was below average, possibly hinting at a subtle residual executive impairment.

In April 2010, a previously healthy 23-year old woman was admitted to our hospital because of a first generalized epileptic seizure. The family of the patient reported about a slight personality change with inappropriate distrustful and jealous behavior that had occurred in previous 4 weeks before admission. Neurological examination was unremarkable and there were no signs of a marked neuropsychiatric or mental disorder. Cerebral magnetic resonance imaging (MRI) was normal. CSF examination revealed a pleocytosis of 72 white blood cells (WBC) /μl, an intrathecal synthesis of immunglobulin M and positive oligoclonal bands (OCBs). All polymerase chain reaction (PCR) -tests against neurotropic viruses including herpes simplex virus 1+2, cytomegaly, Epstein-Barr virus, human herpes virus 6, varicella zoster virus, adenovirus, JC virus, and coxsackie virus remained negative. The electroencephalogram (EEG) showed bi-frontal slowing but no epileptic discharges. During the following 12 days the patient progressively developed a dysexecutive syndrome and exhibited a bizarre and disorganized behavior despite treatment with high doses of valproate (1200 mg/day) and lorazepam (2 mg/day) (Figure 1). Furthermore, the patient developed signs of delusion, pathological distrust and diminished speech production necessitating transfer of the patient to a protected ward. The patients psychopathological symptoms deteriorated further and she developed severe psychosis including mutism, alternating phases of agitation and akinesia as well as fluctuating levels of consciousness. In the third week, the patient developed right-sided orofacial myocloniform dyskinesia and eye-movement abnormalities. The patient was severely impaired on neuropsychological testing and ultimately unresponsive (Figure 2). While cerebral MRI and CSF remained largely unaltered, multiple EEG investigations showed severe bi-hemispheric slowing. At that time, anti-NMDAR antibodies in serum and CSF (both 1:2560) and an ovarian tumor were detected. Antiinflammatory treatment with 1000 mg methylprednisolone per day was initiated. After transfer to the intensive care unit, the patient received five cycles of plasma exchange. To prevent a rebound, intravenous immunglobulins (30 g/day for 3 days) were administered. For long-term B-cell depletion, rituximab at a dose of 1000 mg was given. Also, the right side ovarian tumor was surgically removed. Histology revealed a mature teratoma without microscopic structures of nervous tissue. Intravenous methylprednisolone treatment was followed by oral therapy with 75 mg/day (Figure 1).

Thereafter, the patient improved continuously. Five weeks after admission, the patient was fully orientated and showed an adequate communication. Physical mobilization was possible. Rare periods of reduced consciousness with agitation and a constant subtle delusion with distrusting behavior still remained. CSF control investigation showed normalized parameters (WBC <1/μl, OCB negative) and a reduction of the anti-NMDAR antibody titer in serum to 1:320 and in CSF to 1:100.

At follow-up investigation three months later, the patient did not present any neurological or psychiatric abnormalities. Neuropsychological examination revealed a significant improvement (Figure 2). CSF parameters were normal and NMDAR antibodies had declined further to titers of 1:100 in serum and to 1:10 in CSF. Antipsychotic medication was tapered, but antiepileptic therapy with 2000 mg levetiracetam was recommended for further 6 months. She continued her academic study of psychology.

## Methods

### Measurement of NMDAR antibody titers

Anti-NMDAR antibodies in both, CSF and serum, were measured by antigen-antibody-reaction with indirect immunofluorescence on NR1-transfected Human Embryonic Kidney cells (HEK cells) [6] as well as rat hippocampal and cerebellar slices (HEK cells and all slices from Euroimmun, Lübeck, Germany). The patient has given written informed consent to using the CSF samples for scientific purpose and publication of the case details. Ethics approval by the ethics committee (Institutional Review Board) of Düsseldorf University was obtained (3785). Anti- NMDAR antibodies were measured in patients´ samples of CSF and serum taken four days and three, five and 18 weeks after admission.

### Polymerase chain reaction (PCR) -tests against neurotropic viruses

PCR-tests for neurotropic viruses were performed with the standard technique “TaqMan realtime” and revealed no presence of herpes simplex virus 1+2, cytomegalovirus, Epstein-Barr virus, human herpes virus 6, varicella zoster virus, adenovirus, JC virus, and coxsackie virus in the cerebrospinal fluid.

### Cell culture

Cortical cells were freshly prepared from embryonic day 18 Wistar rats. Cell preparation and animal care were performed in compliance with the German Animal Protection law (State Office, Environmental and Consumer Protection of North Rhine-Westphalia). Permission/Ethic approval was granted by the Institutional Review Board of Düsseldorf University (O/5/2009). Dissociated cortical cells were isolated and collected in ice cold N2 medium (Invitrogen, Karlsruhe, Germany) and trypsinized for 10 minutes. After dual centrifugation for 1 minute at 2000 and 5 minutes for 1500 repeats per minute, cells were resuspended in N2-supplemented Neurobasal medium (Invitrogen) containing 5% fetal calf serum (Fisher Scientific, Schwerte, Germany) and plated at a density of 150 × 10^3^/cm^2^ onto poly-D-lysine- and laminin-coated MEAs and cultured in a humidified atmosphere (5% CO2 / 95% air) at 37°C. Medium was replaced twice a week. After approximately 3 weeks in vitro, cultures developed a dense synaptically connected neuronal network and reached stable electrophysiological properties including population bursting. Functional neuronal networks were used for recordings when they reached a minimum bursting activity of 20 bursts per minute. MEA experiments were performed between 26 and 64 days in vitro (see below), a time window of preserved synchronous population bursting. Earlier immunocytochemical experiments of identical cell preparations [11] demonstrated that neural population consists of glial fibrillary acid protein-positive astrocytes, vesicular glutamate transporter 2 -positive glutamatergic neurons and γ-aminobutyric acid-positive neurons. Extensive immunocytochemical control experiments for this and other studies confirmed the above mentioned cellular phenotypes which were stable along multiple preparations. For this study, 3 different preparations were used and randomly assigned to the experiments.

### MEA measurements and experimental design

**Figure 3:**
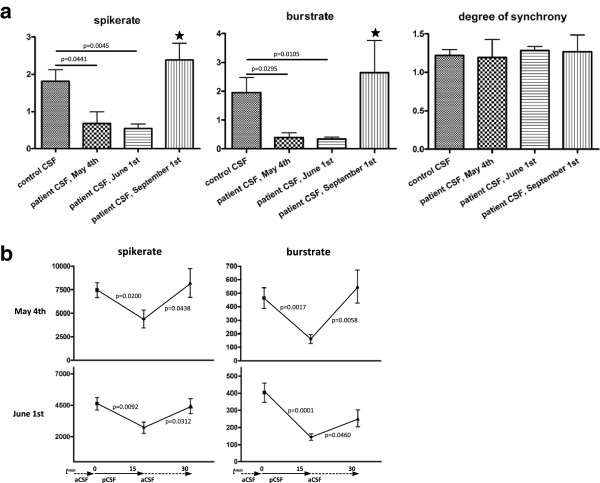
**The microelectrode arrays.** In **a**), diagrams illustrate effects of cCSF and pCSF collected at different time points during the course of the disease. Stars illustrate significances against CSF taken 4 days after admission (May 1^st^) or five weeks after admission (June 1^st^). In **b**), diagrams illustrate quick effects of cCSF (left) or pCSF (middle and right) in comparison to aCSF over a time course of 45 minutes (15 minutes in aCSF, 15 minutes in pCSF and 15 minutes again in aCSF). Bars represent standard error of the mean.

To investigate the potential effect of the patients´ CSF (pCSF) on electrophysiological neuronal network parameters like global network activity and the synchrony of population bursts, pure pCSF of 3 different time points during the course of the disease was administered to at least 4 independent microelectrode arrays (MEAs, Multi Channel Systems, Reutlingen, Germany), respectively. Control experiments with independent MEAs (n=4) were performed using CSF of a healthy person (cCSF). In detail, for MEA experiments that were all continuously performed outside the incubator for 30–40 minutes, cell culture medium was first replaced against aCSF (aCSF; 150 mM NaCl, 1 mM CaCl_2_, 3 mM KCl, 1 mM MgCl_2_, 10 mM N-2-hydroxyethylpiperazine-N-2-ethanesulfonic acid, 10 mM glucose, pH 7.4; Sigma, St. Louis, MO). Network activity was then recorded for the next 15 minutes and averaged for one data point. Then, aCSF was replaced against pure pCSF or cCSF and network activity was again measured for 15 minutes and averaged. The pH was adjusted (HEPES buffer) to 7.4 in all solutions (aCSF, pCSF, cCSF) prior to every experiment and remained constant (+/− pH 0.1) for at least 30 minutes outside the incubator. In Figure 3a, relative values of cCSF/aCSF or pCSF/aCSF are given. In Figure 3b, absolute values are given. Experiments on every single MEA were performed in a standardized design (aCSF for 15 minutes – cCSF or pCSF for 15 minutes). For statistical analyses, the paired student t-test was performed and data are presented as mean ± standard error of means. All data analyses have been performed with GraphPadPrism, version 4.0.

To measure the electrophysiological activity of neuronal populations, MEAs with a square grid of 60 planar Ti/TiN electrodes (30-μm diameter, 200-μm spacing) were used. Signals from all 60 electrodes were simultaneously sampled at 25 kHz, visualized and stored using the standard software MC_Rack provided by Multi Channel Systems. Spike and burst detection was performed off-line by custom-built software (Result, Düsseldorf, Germany). Details of data collection are given elsewhere [12]. Temporal synchrony of spike activity across electrodes was determined by binning all spikes into time intervals of 10 milliseconds. For all pairs of active electrodes, the agreement coefficient Cohen’s kappa was calculated, which measures the degree of coincidence of spikes on both electrodes exceeding the chance-expected coincidence assuming uncorrelated spike activity [13]. The average kappa of all electrode pairs was calculated as measure of overall synchrony of a recording. Theoretically, kappa may vary in the range −1 to +1.

## Results

Patient CSF taken 4 days and 5 weeks after admission (both with NMDAR antibody titers of 1:100) induced a significant suppression of global activity markers (spike rate and burst rate) in spontaneously bursting in vitro-cortical neuronal networks in comparison to CSF from a healthy control person (Figure 3a, left and middle diagram). Unfortunately, the CSF sample with antibody titers of 1:2560 (third week after admission) was too small precluding further analysis. CSF from the same patient 18 weeks after admission to hospital, after therapy and in greatly improved clinical condition did not suppress these parameters any longer. The NMDAR antibody titer at this time point was 1:10. Interestingly, the degree of network synchrony was unaltered under the influence of all different pCSF probes (Figure 3a, right diagram). Furthermore, we could demonstrate a quick suppressive effect of pCSF on spikerates within the first 15 minutes after application of pCSF, that was taken five weeks after admission (Figure 3b, right diagram). This investigation was done in comparison to artificial CSF (aCSF) in a paired fashion on the same MEA. Patient CSF taken 4 days after admission only tendentially decreased quickly the spikerate in comparison to aCSF (Figure 3b, middle diagram) showing that the speed of network alterations might vary between pCSF probes that indeed showed identical NMDAR antibody titers but that were taken at different time points during the course of the disease. In contrast, application of CSF of a healthy control (cCSF) typically increased the spikerate (Figure 3b, left diagram).

## Discussion

We present a case with severe paraneoplastic anti-NMDAR encephalitis and complete recovery after surgical removal of the ovarian teratoma and multimodal immunomodulatory therapy. High-dose methylprednisolone treatment, plasma exchange and administration of immunoglobulins rapidly reversed the downhill course and allowed the patient to recover. Most likely, B-cell depletion with rituximab sustained the subsequent complete recovery. Following the complete clinical recovery, OCBs were no longer detectable. Disappearance of OCBs from the CSF after treatment is highly unusual. As NMDAR antibody titers, also at the late time point, were still measurable, it is unlikely that OCBs represented a specific anti-NMDAR immune response.

It was previously found that NMDAR auto-antibodies lead to receptor internalization and, consecutively, a hypo-functional state including decreased NMDAR currents [6]. Here, we were able to show that the patient’s CSF suppresses global activity in functional neuronal networks while synchrony of population bursts remained unaltered. Recently published results demonstrated that modulation at the NMDA receptor can significantly influence in vitro-neuronal network activity (ivNNA) [10,14]. Importantly, NMDAR modulation was shown to inversely influence burst rates and the degree of synchrony of the network which is expressed by the kappa value [14]. Concerning suppression of NMDAR function, the NMDA-receptor antagonist DL-2-Amino-5-phosphono-pentanoic acid (AP-5) can supress the burst rate while preserving the kappa value [10]. This directly illustrates that a hypofunctional state of NMDA receptors exerts complex reactions within a system that depends on a variety of different electrophysiological players. And this suggests that rhythmic activity of neuronal networks can still be present under the influence of suppressing NMDAR auto-antibodies. An electrophysiological constellation combining a hypo-functional state including decreased NMDAR activity with preserved rhythmic network activity might then relate to the clinical hallmarks that comprise psychosis and epileptic seizures illustrating the importance of measuring pharmacological effects in a more complex system that mimics basic features of oscillating brain activity. Although the system used in our study represents artificial in vitro-neuronal network activity, a body of work has described its activity under different extracellular compositions that in summary suggest a physiological-like activity of population bursting under balanced extracellular conditions. Addition of neuroactive substances, e.g. acting on glutamatergic or GABAergic synapses or modulating voltage-dependant ion channels, change that activity which is described in multiple reports [10,12,14,15]. Thus, pathological-like conditions of this artificial in vitro system can be defined.

The pCSF-related effects observed in this study resolved in parallel to clinical improvement and a reduction of the antibody titer to 1:10. We could also demonstrate a fast suppressive effect of pCSF on network activity within the first 15 minutes after pCSF application in comparison to aCSF suggesting that next to NMDAR capping and internalization, pCSF might additionally induce a rapidly evolving dysfunction of neuronal network activity. The reversibility and functional dynamics of neuronal network activity under the influence of pCSF shown here and the course of anti-NMDAR antibody titers in our patient strengthen the notion that anti-NMDAR antibodies mediate this neuropsychiatric syndrome. However, it remains to be elucidated if antibodies can indeed exert such a fast functional effect on NMDA receptors as antibody binding usually needs some time. Our report has several shortcomings: First, we only investigated one pCSF. Second, as a variety of different drugs were administered in the course of the disease, additional drug-related effects cannot be completely excluded. However, as benzodiazepines were administered only during the second collection of lumbar CSF, we consider benzodiazepine effects on neuronal network activity unlikely. Third, we were not able to directly attribute the observed effects to NMDAR autoantibodies as the limited volume of the CSF probes rendered further pCSF-based investigations impossible. Thus, further experiments with a higher number and larger volume of CSF probes from different NDMAR encephalitis patients are needed.

## Conclusion

We described a method to assess functional consequences of pathologically altered CSF specimens from a NMDA receptor encephalitis patient. As new limbic encephalitis antibodies against surface epitops are continuously discovered newly, this method can serve to investigate CSF specimens from so far antibody-negative limbic encephalitis patients.

## Abbreviations

AMPA: α-amino-3-hydroxy-5-methyl-4-isoxazolepropionic acid; aCSF: artificial cerebrospinal fluid; CSF: cerebrospinal fluid; EEG: electroencephalography; GABA: gamma-aminobutyric acid; HEK cells: human embryonic kidney cells; ivNNA: in vitro neuronal network activity; MEA: microelectrode array; mEPSCs: miniature excitatory post synaptic currents; MRI: magnetic resonance imaging; NMDA: N-methyl-D-aspartate; NMDAR: N-methyl-D-aspartate-receptor; OCB: oligoclonal bands; PCR: polymerase chain reaction; pCSF: patient´s cerebrospinal fluid; WBC: white blood cells.

## Authors’ contribution

SUJ, DS and RJS were responsible for the clinical course of the patient. MD and SU.J wrote the manuscript. CW was responsible for the neuropsychological examinations and corresponding parts in the manuscript. SF, KQ, SI and MD are responsible for the examination of the CSF by the MEA technology. H-PH, RS and MD revised the manuscript for intellectual contents. All authors read and approved the final manuscript.

## References

[B1] Dalmau J Tuzun E Wu HY Masjuan J Rossi JE Voloschin A Baehring JM Shimazaki H Koide R King D Paraneoplastic anti-N-methyl-D-aspartate receptor encephalitis associated with ovarian teratoma Ann Neurol 2007 61 1 25 36 10.1002/ana.21050 17262855 PMC2430743

[B2] Dalmau J Gleichman AJ Hughes EG Rossi JE Peng X Lai M Dessain SK Rosenfeld MR Balice-Gordon R Lynch DR Anti-NMDA-receptor encephalitis: case series and analysis of the effects of antibodies Lancet Neurol 2008 7 12 1091 1098 10.1016/S1474-4422(08)70224-2 18851928 PMC2607118

[B3] Dalmau J Lancaster E Martinez-Hernandez E Rosenfeld MR Balice-Gordon R Clinical experience and laboratory investigations in patients with anti-NMDAR encephalitis Lancet Neurol 2011 10 1 63 74 10.1016/S1474-4422(10)70253-2 21163445 PMC3158385

[B4] Florance NR Davis RL Lam C Szperka C Zhou L Ahmad S Campen CJ Moss H Peter N Gleichman AJ Anti-N-methyl-D-aspartate receptor (NMDAR) encephalitis in children and adolescents Ann Neurol 2009 66 1 11 18 10.1002/ana.21756 19670433 PMC2826225

[B5] Day GS High SM Cot B Tang-Wai DF Anti-NMDA-receptor encephalitis: case report and literature review of an under-recognized condition J Gen Intern Med 26 7 811 816 21318640 10.1007/s11606-011-1641-9 PMC3138579

[B6] Hughes EG Peng X Gleichman AJ Lai M Zhou L Tsou R Parsons TD Lynch DR Dalmau J Balice-Gordon RJ Cellular and synaptic mechanisms of anti-NMDA receptor encephalitis J Neurosci 2010 30 17 5866 5875 10.1523/JNEUROSCI.0167-10.2010 20427647 PMC2868315

[B7] Lau CG Zukin RS NMDA receptor trafficking in synaptic plasticity and neuropsychiatric disorders Nat Rev Neurosci 2007 8 6 413 426 17514195 10.1038/nrn2153

[B8] Krystal JH Karper LP Seibyl JP Freeman GK Delaney R Bremner JD Heninger GR Bowers MB Jr Charney DS Subanesthetic effects of the noncompetitive NMDA antagonist, ketamine, in humans. Psychotomimetic, perceptual, cognitive, and neuroendocrine responses Arch Gen Psychiatry 1994 51 3 199 214 10.1001/archpsyc.1994.03950030035004 8122957

[B9] Weiner AL Vieira L McKay CA Bayer MJ Ketamine abusers presenting to the emergency department: a case series J Emerg Med 2000 18 4 447 451 10.1016/S0736-4679(00)00162-1 10802423

[B10] Otto F Illes S Opatz J Laryea M Theiss S Hartung HP Schnitzler A Siebler M Dihne M Cerebrospinal fluid of brain trauma patients inhibits in vitro neuronal network function via NMDA receptors Ann Neurol 2009 66 4 546 555 10.1002/ana.21808 19847897

[B11] Schwarz CS Ferrea S Quasthoff K Walter J Gorg B Haussinger D Schnitzler A Hartung HP Dihne M Ammonium chloride influences in vitro-neuronal network activity Exp Neurol 235 1 368 373 22421534 10.1016/j.expneurol.2012.02.019

[B12] Illes S Theiss S Hartung HP Siebler M Dihne M Niche-dependent development of functional neuronal networks from embryonic stem cell-derived neural populations BMC Neurosci 2009 10 93 10.1186/1471-2202-10-93 19660102 PMC2733139

[B13] Woolson R CW: Statistical Methods for the Analysis of Biomedical Data 2002 New York: Wiley-Interscience

[B14] Schwarz CS Ferrea S Quasthoff K Walter J Gorg B Haussinger D Schnitzler A Hartung HP Dihne M Ammonium chloride influences in vitro-neuronal network activity Exp Neurol 2012 235 1 368 373 10.1016/j.expneurol.2012.02.019 22421534

[B15] Illes S Fleischer W Siebler M Hartung HP Dihne M Development and pharmacological modulation of embryonic stem cell-derived neuronal network activity Exp Neurol 2007 207 1 171 176 10.1016/j.expneurol.2007.05.020 17644089

